# Nutrient Levels, Bioactive Metabolite Contents, and Antioxidant Capacities of Faba Beans as Affected by Dehulling

**DOI:** 10.3390/foods12224063

**Published:** 2023-11-08

**Authors:** Yu-Mi Choi, Hyemyeong Yoon, Myoung-Jae Shin, Sukyeung Lee, Jungyoon Yi, Young-ah Jeon, Xiaohan Wang, Kebede Taye Desta

**Affiliations:** 1 National Agrobiodiversity Center, National Institute of Agricultural Sciences, Rural Development Administration, Jeonju 54874, Republic of Korea; 2 International Technology Cooperation Center, Technology Cooperation Bureau, Rural Development Administration, Jeonju 54875, Republic of Korea

**Keywords:** antioxidant activity, dehulling, faba beans, metabolite contents, nutrition level, *Vicia faba*

## Abstract

Legume dehulling often removes anti-nutrients while improving nutritional quality. However, the process may reduce the levels of other health-promoting metabolites. This study investigated the effect of dehulling on major nutrients, bioactive metabolites, and antioxidant activities using 22 faba bean cultivars typically grown in different parts of the world. The faba bean cultivars differed significantly in all the parameters assessed. Crude fiber (CFC), dietary fiber (DFC), crude protein, and crude fat contents were in the ranges of 5.24–10.56, 16.17–25.15, 19.83–30.90, and 0.79–1.94% in the whole seeds and 0.96–1.59, 4.14–9.50, 22.47–36.61, and 1.13–2.07% in the dehulled seeds, respectively. Moreover, fatty acids including palmitic acid, stearic acid, oleic acid, linoleic acid, and linolenic acid, bioactive metabolites including total phenol (TPC), total saponin (TSC), and total tannin (TTC) contents, and antioxidant activities including ABTS^•+^-scavenging activity, ferric antioxidant power (FRAP), and DPPH^•^-scavenging activity also showed significant variations. Dehulling significantly reduced DFC (55.09–79.30%), CFC (69.61–87.52%), and TTC (1.70–66.99%) in all the faba bean cultivars while increasing total protein content (9.31–17.69%). Dehulling also increased the total fat content (3.02–48.13%) in all the cultivars except Giant Three Seeded, a Japanese cultivar, which showed a 12.62% decrease. In contrast, dehulling exhibited varying results on fatty acids, TPC, TSC, and antioxidant activities among the faba bean cultivars. Accordingly, three cultivars: Primus from Hungary, Levens Marschbohne from Germany, and Ascott from France, exhibited simultaneous increases in nutritional levels after dehulling. Domasna-2 from Macedonia, Abawi# 1 from Peru, Seville from the United Kingdom, and Large Mazandran from Iran, on the other hand, exhibited marked reductions in nutritional levels, functional metabolites, and antioxidant activities. In general, our findings indicated that dehulling reduces crude fiber, dietary fiber, and tannin levels while increasing protein and fat contents in faba beans. However, fatty acids, phenolic content, and antioxidant activity may not be equally affected by dehulling and, therefore, specific genotypes should be inspected.

## 1. Introduction

Faba bean (*Vicia faba* L.) is a leguminous plant that grows all over the world. Its beans are well known for their health-promoting metabolite compositions. Specifically, faba beans are high in dietary fibers, essential minerals, carbohydrates, and vitamins [1,2,3]. Moreover, they contain both saturated and unsaturated fatty acids, which contribute to their nutritional composition and health benefits. They are also ideal sources of essential amino acids, making them an excellent source of protein for vegetarians. As a result, faba beans can be used instead of soybean meal, a prominent protein source for both humans and livestock, minimizing reliance on a single source [4,5]. In addition to these nutritional components, faba beans contain several other bioactive secondary metabolites, particularly polyphenols and phytosterols, which contribute to their health benefits. Because of these broad classes of metabolites, faba beans exhibit a variety of bioactivities, including antioxidant, anti-inflammatory, and anti-cancer characteristics [6,7]. Moreover, faba beans have a low glycemic index, which can help manage blood sugar levels and lower the risk of developing diabetes [8]. 

Faba beans, on the other hand, contain anti-nutrient components such as tannins, lectins, phytic acid, vicine, and convicine, which limit the bioavailability and digestibility of their nutrients [9]. The anti-nutrient components in faba beans are the primary obstacles inhibiting the broad use of faba bean genotypes in food enterprises and breeding technologies [10,11]. Researchers have devised various processing strategies to minimize the levels of anti-nutrient components in legumes in general. Some of the processing processes thought to minimize and/or eliminate anti-nutrients are dehulling, germination, cooking, and roasting [11,12,13,14]. However, each of these approaches has its own set of disadvantages. Dehulling, the process of removing the seed coats (hulls), for example, has been used to reduce the levels of some anti-nutrient factors and at the same time maximize some of the nutritional levels of legumes [15]. Despite such positive outcomes, dehulling also results in the reduction of the concentrations of other health-promoting metabolites. This is especially relevant because the distribution of metabolites differs between the different seed parts. Some metabolites are concentrated in the seed coats, while others are concentrated in the cotyledon and/or whole seeds. Even the distribution of metabolites across different seed portions varies between genotypes, further complicating matters [16,17]. These signify that dehulling and other specific processing methods may not be suitable for all genotypes and, hence, establishing their effectiveness in diverse faba bean varieties is crucial [16]. 

Previously, several studies have attempted to evaluate the effect of dehulling on faba bean nutrients, metabolites, and biological activities. In a study conducted three decades ago, Youssef et al. [18] discovered that dehulled seeds had elevated levels of Zn and K in comparison to whole seeds. Nevertheless, the same research indicated a decrease in the levels of Fe, Mn, Cu, and Na in dehulled seeds. In four separate studies, Luo et al. [2,19,20,21] showed the effect of dehulling on mineral contents and on how the process could reduce anti-nutrient factors. In a more recent study by Rajhi et al. [22], whole seeds were found to have better antioxidant activities compared to dehulled seeds. However, removing the hulls enhanced the presence of volatile molecules in the seeds. In a comparable study, dehulled faba beans were found to be superior in protein contents and non-starch polysaccharides compared to whole seeds [3,23]. Despite such research advancements, most studies focused on a single faba bean genotype or a certain class of chemicals. Furthermore, the effect of dehulling on other faba bean metabolites, such as individual fatty acids and polyphenols, and biological activities remains underexplored. Overall, studying the effect of dehulling on different faba bean genotypes could provide a better understanding of its impact on metabolite compositions and biological activities [16]. Such understandings ensure that faba beans are used by consumers and the food industry in ways that maximize their health advantages while reducing potential negative repercussions [24]. Furthermore, examining the effect of dehulling on different faba bean varieties aids in the selection of genotypes that are better adapted to this specific processing step without losing their nutritional benefits [10,15]. The aim of this study was therefore to evaluate the effect of dehulling on the contents of major nutrients (crude fiber, dietary fiber, total protein, and total fat), fatty acids (palmitic acid, stearic acid, oleic acid, linoleic acid, and linolenic acid), functional metabolites (total phenolic content, total tannin content, and total saponin contents), and antioxidant activities (DPPH^•^-scavenging activity, ferric reducing antioxidant power (FRAP), and ABTS^•+^-scavenging activity) using 22 faba bean cultivars that are widely grown in fourteen different countries. 

## 2. Materials and Methods

### 2.1. Chemicals and Reagents

All the chemicals and reagents used in this study were of analytical grade and used as obtained. Ethanol and sulfuric acid were purchased from Fisher Scientific (Pittsburgh, PA, USA). All the other chemicals and reagents were ordered from Sigma-Aldrich (St. Louis, MO, USA). 

### 2.2. Seed Material Collection, Cultivation, and Preparation

The 22 faba bean cultivars were accessed from our gene bank at the National Agrobiodiversity Center, Rural Development Administration (Jeonju, Republic of Korea). The faba beans were originally collected from different countries where they are widely cultivated. Abawi# 1 is from Peru, Aguadulce, Brocal, Muchamiel, and Tempranas De Machamiel are from Spain, Algerian and Large Mazandaran are from Iran, Alicante, Ethiopia 530, and Yavneh are from Israel, Ascott is from France, Domasna-1, Domasna-2, and Strumicka are from Macedonia, Giant Three Seeded is from Japan, Levens Marschbohne is from Germany, MMR-KJT-2010-K161716 is from Myanmar, NPL-JSW-2003-65 is from Nepal, Pirkkonen is from Sweden, Primus is from Hungary, Seville is from the United Kingdom, and Zborovicki is from the Czech Republic (Table S1). The faba bean cultivars were cultivated under similar conditions on an experimental farm located at our center (Jeonju, Republic of Korea) between April and July of 2021. Matured seeds were hand-harvested and samples from each cultivar were freeze-dried (LP500 freeze dyer, ilShinBioBase, Dongducheon, Republic of Korea). Then, the seed coats were hand-peeled, and the whole seeds and dehulled seeds samples were powdered using an electric grinder. Powdered samples were passed through sieves of 500 µm mesh size and stored at −20 °C pending extraction and further analysis.

### 2.3. Determination of Nutritional Components 

The contents of crude fiber, dietary fiber, crude protein, and crude fat were determined using standard analytical methodologies proposed by AOAC [25]. Briefly, crude fiber content was determined using a fiber analyzer (FOSS, Hillerod, Denmark) following the modified Henneberg and Stohmann method, whereas dietary fiber content was determined by an enzymatic–gravimetric assay using an Analytical Fibertec E-1023 System (FOSS, Hillerod, Denmark). Likewise, crude protein content was determined using the Kjeldahl method and calculated as *N* × 6.25. The Soxhlet extraction method, using a Soxtec800 extractor (FOSS, Hillerod, Denmark) and *n*-hexane as a solvent, was used to determine the crude fat content. In each case, whole seed and dehulled seed samples of each cultivar were prepared and analyzed in triplicate. The results are reported as percentages based on dried seed weight. 

### 2.4. Fatty Acid Analysis by Gas Chromatography–Flame Ionization Detector (GC-FID) Instrument 

For fatty acid analysis, fatty acid methyl ester (FAME) derivatives were synthesized using a direct methylation technique as reported in a previous study [26]. In brief, 0.2 g of powdered sample was put in a 10 mL round-bottom glass tube with a screw cap. Then, 680 µL of a solvent mixture consisting of methanol, benzene, 2,2-dimethoxypropane, and sulfuric acid in the ratio of 39:20:5:2 was added followed by the addition of 400 µL of *n*-heptane. The mixture was vortexed and extraction was achieved in a shaking water bath set at 80 °C for 2 h. The mixture was then taken off and cooled at 25 °C followed by centrifugation (3134× *g*, 15 min). Finally, the upper *n*-heptane layer containing FAMEs was collected, filtered, and readied for GC-FID analysis. A pre-optimized QP2010 GC-FID instrument (Shimadzu, Kyoto, Japan) outfitted with an HP-INNOWAX column (30 m × 0.250 mm, 0.25 µm) was used for fatty acid analysis. The sample injection volume was 1 µL at a split ratio of 50:1. Helium, at a flow rate of 1.5 mL/min, was used as a carrier gas. The initial column temperature was set at 100 °C and gradually increased to 170 °C at a rate of 60 °C/min with a holding time of 1 min. The temperature was then raised to 240 °C with a 6.5 °C/min ramp and held for another 1 min. The total analysis time was 16.4 min. The detector and injection port were set at 250 °C. LabSolution software version 5.92 (Shimadzu, Kyoto, Japan) was used to control the analysis process and to analyze the acquired GC chromatograms. The fatty acids were identified using the retention times of the corresponding external standards. Fatty acid contents were calculated as the relative percent of the total fatty acid using area peaks.

### 2.5. Determination of Total Tannin, Saponin, and Phenol Contents and Antioxidant Activities

For the determination of total tannin, total saponin, total phenol, and antioxidant activities, extracts were prepared according to the method reported by Boudjou et al. [27]. In brief, 0.5 g of powdered sample, in triplicate, was mixed with 5 mL of aqueous ethanol (80%) in a 15 mL extraction tube. The mixture was vortexed to mix and sonicated for 45 min in the dark at 25 °C. Then, the mixture was taken off, centrifuged (3134× *g*, 10 min), and the upper layer was retained. The residue was re-extracted once more using 2.5 mL of the solvent. The combined supernatant was then used for the determination of total metabolite contents and antioxidant activities as briefed below. In each case, absorbance was measured using an Eon Microplate Spectrophotometer (Bio-Tek, Winooski, VT, USA).

#### 2.5.1. Determination of Total Tannin Content

Total tannin content (TTC) was determined using the vanillin–HCl method as proposed by Price et al. [28] with slight modification. In brief, 100 µL of sample extract was mixed with 200 µL of a vanillin–HCl reagent which was prepared by mixing equal parts methanol solutions of 8% HCl and 1% vanillin. Then, the mixture was incubated for 20 min at 25 °C in the dark, followed by absorbance measurement at 500 nm. Catechin (0.05–0.70 mg/mL) was used as a standard to plot calibration curves (R^2^ > 0.999), and the TTC was computed as milligrams of catechin equivalents per gram of dried seed weight (mg CE/g). 

#### 2.5.2. Determination of Total Saponin Content

The vanillin–sulfuric acid assay was used for the determination of total saponin content (TSC) following a previously described method with slight modification [29]. Briefly, 25 µL sample extract was mixed with an equal amount of freshly prepared 8% vanillin (*w*/*v* in ethanol). Then, 250 µL of 72% sulfuric acid (*v*/*v* in water) was added, and the mixture was incubated in a water bath at 60 °C for 10 min. Then, the mixture was taken off and cooled in an ice bath for 15 min before the absorbance was measured at 544 nm. Diosgenin (0.10–1.50 mg/mL) was used to plot calibration curves (R^2^ > 0.999) and the TSC was then calculated as milligrams of diosgenin equivalent per gram of dried sample (mg DE/g).

#### 2.5.3. Determination of Total Phenolic Content and Antioxidant Activities

Total phenolic content (TPC) and antioxidant activities were determined according to our recently reported protocol [30]. In summary, TPC was estimated using the Folin–Ciocalteu method and reported as milligrams of gallic acid equivalents per gram of dried seed weight (mg GAE/g) using gallic acid as a standard. The antioxidant capacities, including DPPH^•^-scavenging activity and ferric-reducing antioxidant power (FRAP), were estimated using in vitro colorimetric assays, and each was reported as milligrams of ascorbic acid equivalents per gram of dried seed weight (mg AAE/g) using ascorbic acid as a standard. For ABTS^•+^-scavenging activity, Trolox was used as standard and the values were reported as milligrams of Trolox equivalents per gram of dried seed weight (mg TE/g).

### 2.6. Statistical Analysis

All measurements and analyses in this study were conducted in triplicate regardless of sample type and results are expressed as mean ± standard deviation (SD). Statistical analysis was conducted by analysis of variance (ANOVA) followed by Duncan’s multiple range test using xlstat software version 2019.2.2 (Lumivero, CO, USA). Box plots and principal component and Pearson’s correlation analyses were computed using *R* software (version 4.0.2, r-project). 

## 3. Results and Discussion 

### 3.1. Crude and Dietary Fiber Contents

**Figure 1:**
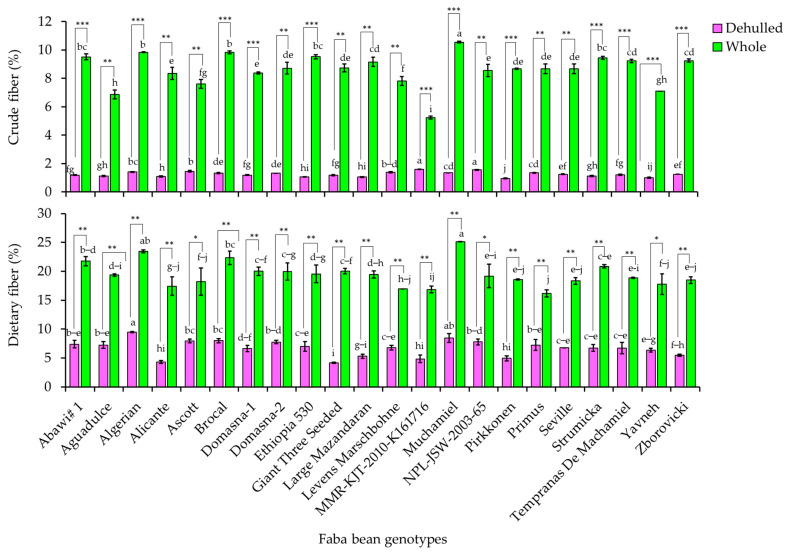
Variations of crude and dietary fiber contents across dehulled and whole seed faba bean genotypes. Letters on bars in a category indicate significantly different means between faba bean cultivars. * *p* < 0.05, ** *p* < 0.01, *** *p* < 0.001 between whole and dehulled seed samples of a specific faba bean cultivar.

The crude fiber and dietary fiber contents in the whole seeds and dehulled seeds of the 22 faba bean cultivars are presented in Figure 1. The numerical values can be viewed in Table S1 (Supplementary Materials). The crude fiber content was in the ranges of 5.24–10.56 and 0.96–1.59%, while the dietary fiber content was in the ranges of 16.17–25.15 and 4.14–9.50% in the whole seeds and dehulled seeds, respectively. Previously, several studies estimated the levels of crude fiber and dietary fiber contents in faba beans, and variable results were reported. For example, Labba et al. [31] reported a total dietary fiber content varying from 11.37 to 16.59% across 15 faba bean cultivars produced in Sweden. In another study, raw seeds of three faba bean varieties grown in Mexico were shown to have a much lower dietary fiber content ranging from 1.54 to 1.98% [13]. A dietary fiber content as high as 27.50% and a crude fiber content as high as 5.27% were also reported [32,33]. Other studies also found a wide range of fiber contents in different faba bean genotypes, demonstrating the influence of genetic variety, growing conditions, and post-harvest handling methods [19,34]. Overall, the dietary and crude fiber contents observed in our study fall within previously reported ranges [1].

As shown in Figure 1, significant variations of crude and dietary fiber contents in both the whole seeds and dehulled seeds (*p* < 0.05) were observed between the faba bean cultivars. Among the 22 faba beans, Muchamiel displayed the highest crude fiber and dietary fiber contents in the whole seeds. Similarly, Primus and Giant Three Seeded had the lowest dietary fiber contents in whole and dehulled seeds, respectively. MMR-KJJ-2010-K161716 had the highest crude fiber content in the dehulled seeds but the lowest crude fiber content in the whole seeds. Algerian and Perikkonen had the highest dietary fiber and the lowest dietary fiber contents in the dehulled seeds, respectively. In general, our results demonstrated a wide range of crude fiber and dietary fiber contents across the 22 faba bean cultivars. These results demonstrate the effect of genetic variance on fiber contents as well as the variations of fiber levels across faba bean seed sections [19,34]. Fibers are important components in faba beans and play a crucial role in the prevention of cardiovascular diseases, constipation, and the risk of colon cancer [1,34]. Therefore, the cultivars including Muchamiel, Algerian, and Brocal could be ideal resources owing to their high levels of fibers.

The effect of dehulling was also statistically analyzed, and the process significantly lowered both crude fiber and dietary fiber levels in all the faba bean cultivars (Figure 1). In the whole population, dehulling resulted in a 7-fold decrease in crude fiber and a 3-fold reduction in dietary fiber (Figure S1, Table S1). The biggest drop in crude fiber was found in Pirkkonen (88.96%), while the lowest decrease was observed in MMR-KJT-2010-K161716 (69.61%). In contrast, Giant Three Seeded had the greatest drop in dietary fiber (79.30%), while Primus had the smallest (55.09%). Previous studies have investigated the effect of dehulling on fiber contents in a range of legumes, including lentils, chickpeas, and cowpeas, among others, with the majority of studies resulting in reduced fiber contents after dehulling [35,36]. Compared to other nutritional components, the effect of dehulling on faba bean fibers has received less attention. Despite this, a few studies have indicated that dehulling also reduces fiber levels in faba beans, which supports our findings [18,20]. Overall, the differences in the crude fiber and dietary fiber contents between whole and dehulled seeds indicate the variable distribution of fibers in the different parts of faba bean seeds. Furthermore, the significant decreases in the crude fiber and dietary fiber contents upon dehulling indicate that the seed coat is an important component of faba beans as a source of fiber [1]. Therefore, consuming whole faba beans is highly recommended to obtain a much better fiber level. 

### 3.2. Crude Protein and Crude Fat Contents

**Figure 2:**
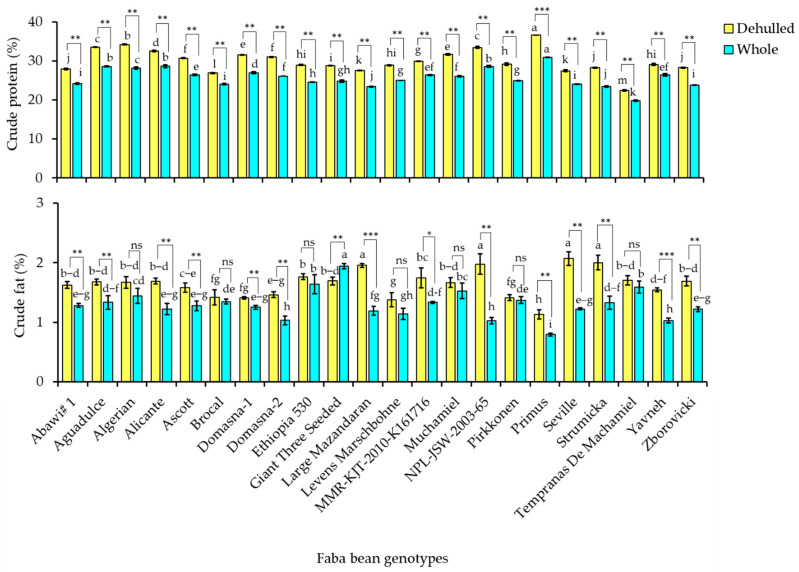
Variations of crude protein and crude fat contents across dehulled and whole seed faba bean genotypes. Letters on bars in a category indicate significantly different means between faba bean cultivars. * *p* < 0.05, ** *p* < 0.01, *** *p* < 0.001 between whole and dehulled seed samples of a specific faba bean cultivar. ^ns^ Not significant.

Figure 2 depicts the total protein and total fat contents in the whole and dehulled seeds of the 22 faba bean cultivars. The crude protein content ranged from 19.83 to 30.90% in the whole seeds and from 22.47 to 36.61% in the dehulled seeds. Similarly, the crude fat content in the whole and dehulled seeds was in the ranges of 0.79–1.94% and 1.13–2.07%, respectively. Several prior studies on the crude protein and fat levels of faba bean varieties have yielded comparable results. For example, protein and fat levels in the raw seeds of five faba bean varieties ranged from 31.28–33.30% and 1.18–1.26%, respectively [37]. In other studies, total protein levels ranged from 26.59 to 35.17% in three Mexican faba bean varieties and from 22.7 to 28.3% in fifteen Swedish faba bean cultivars [13,31]. A recent review by Rahate et al. [24] revealed that crude fat content in faba bean flour could range from 1.5 to 2.0% which was close to our findings. As shown in Figure 2, significant variations in crude protein and crude fat contents were observed among the cultivars (*p* < 0.05). Primus yielded the highest crude protein content among the 22 cultivars, while Tempranas De Machamiel yielded the lowest in both whole and dehulled seeds. The whole seeds of Giant Three Seeded and the dehulled seeds of Seville had the highest crude fat levels. In contrast to its high protein level, Primus had the lowest fat content in both whole and dehulled seeds. Similar to Primus, the majority of faba bean cultivars showed contradictory amounts of crude protein and crude fat, which could be attributed to their distinct biosynthetic pathways. 

Protein and fat levels in faba beans, like other dietary components, are affected by various processing methods, as well as genetic and environmental factors, resulting in a wide range of values [1]. The effect of dehulling on both crude protein and crude fat contents is displayed in Figure 2 and Figure S1 (Supplementary Materials). The numeric values are provided in Table S1. In contrast to its effect on fiber contents, dehulling significantly increased the crude protein level in all the faba bean cultivars. With an increase of 17.69%, Algerian showed the highest increment, while Yavneh showed the lowest at 9.31%. The crude fat content followed a similar pattern, with all faba bean cultivars except Giant Three Seeded having a higher fat level in their dehulled seeds than in their whole seeds. Except in seven cultivars, dehulled seeds had significantly higher level of crude fat content than the whole seeds (Figure 2). Large Mazandaran, Seville, and NPL-JSW-2003-65 saw the largest increases, with 39.10, 40.80, and 48.13%, respectively. Pirkkonen, on the other hand, showed the smallest increment with only 3.02%, followed by Brocal, which increased by 4.79%. In support of our results, Ghavidel and Prakash [35], for example, found a significant rise in both crude protein and crude fat content in chickpea, lentil, and cowpea genotypes. Surprisingly, Guajardo-Flores et colleagues [38] discovered a considerable fall in the level of total protein after dehulling of a Mexican black bean (*Phaseolus vulgaris* L.) cultivar, with no effect on total fat. Several studies on faba beans found that dehulled seeds had higher total fat and total protein levels than whole seeds [15]. Our findings further ascertain that the levels of crude fat and crude protein of faba beans can be increased upon dehulling the seed coats. As a result, it is recommended that dehulled seeds be used as protein and fat sources. Furthermore, the faba bean cultivars including Algerian, Muchamiel, Large Mazandaran, Seville, and NPL-JSW-2003-65 that exhibited a high protein and fat content in their dehulled seeds could be major sources of nutrition.

### 3.3. Fatty Acid Contents

Legumes are thought to be important sources of fatty acids, mainly omega-3, that play a significant role in preventing various diseases related to blood pressure. However, the fatty acid components of some legume genotypes, including faba beans, and how processing methods affect their levels are still underexplored. Therefore, further studies are required to amplify their application for human nutrition [39,40]. In this study, the fatty acid contents in the whole seeds and dehulled seeds of the 22 faba bean cultivars were examined using GC-FID as previously described. A total of five fatty acids with varying oxidation states were found in all the cultivars, and their contents are presented in Table 1. The contents of palmitic acid and stearic acid, the two saturated fatty acids, were in the ranges of 14.93–18.83% and 2.22–3.26% in the whole seeds and 15.51–19.09% and 1.86–3.26% in the dehulled seeds, respectively. Similarly, the contents of oleic acid, linoleic acid, and linolenic acid were in the ranges of 22.97–39.54, 39.53–54.44, and 1.80–3.11% in the whole seeds and 22.43–39.50, 40.09–55.28, and 1.81–3.13% in the dehulled seeds, respectively. As reported in several earlier studies, PA was the dominant saturated fatty acid, while LA was the dominant unsaturated fatty acid in all cultivars, regardless of sample type [41,42,43]. Previous studies have assessed fatty acid levels in several faba bean genotypes, and the reported values vary widely due to differences in genotype, growth circumstances, and post-harvest processing, among other factors [17,44]. The fatty acid contents found in our study are comparable to those found by Yoshida et al. [43], who reported palmitic, stearic, oleic, linoleic, and linolenic acid contents in the ranges of 14.0–23.50, 2.00–2.40, 22.60–23.80, 50.80–54.30, and 3.80–5.10%, respectively, across four faba bean cultivars. In comparison to our findings, another study by Ryu et al. [42] found greater PA (16.90–20.80%) and LA (45.70–63.6%) contents but lower SA (0.0–3.1%) and OA (5.5–12.7%) contents among ten faba bean genotypes.

The levels of the fatty acids varied significantly among the faba bean cultivars, as illustrated in Table 1 (*p* < 0.05). Large Mazandaran had the highest PA and LLA in both whole and dehulled seeds, whereas Zborovicki and Primus had the lowest PA and LLA in both samples, respectively (*p* < 0.05). Similarly, in both whole and dehulled seeds, Ascott had the lowest SA while NPL-JSW-2003-65 had the highest. NPL-JSW-2003-65 also had the lowest OA in its whole seeds. Seville had the maximum OA and the lowest LA in both whole and dehulled seeds, while Levens Marschbohne had the lowest OA in the dehulled seeds and the highest LA in both whole and dehulled seeds. Many of the faba bean cultivars, including these latter cultivars, had contradictory LA and OA values. This could be explained by the effects of several desaturase enzymes, which govern the interconversion of these fatty acids [39]. In general, our findings demonstrated a wide range of fatty acid levels in the faba bean cultivars, with total unsaturated fatty acid (TUFA) content being higher than total saturated fatty acid (TSFA) content regardless of sample type. The presence of large quantities of unsaturated fatty acids in legumes is desirable due to their health advantages [41,44]. In this aspect, faba beans have unique characteristics compared to other legumes. For example, a recent study found that a significant amount of LA found in faba beans could protect against coronavirus disease (COVID-19) infection [45]. Another study suggested that including faba beans in the diet of broiler chickens could enhance the fatty acid composition and improve the quality of meat in the breast muscles [46]. Despite such benefits, unsaturated fatty acids also affect the shelf life and stability of faba bean oils because of their vulnerability to oxidation [44]. Seville, which had the lowest double bond index (DBI) in both its whole and dehulled seeds, could be a good source of oil with a long shelf life and stability (Table S1). The effect of dehulling on each of the fatty acids was also statistically investigated, as shown in Table 1. Dehulling lowered the levels of SA and OA in the majority of faba bean varieties, but the other fatty acids had mixed results. These findings could imply that dehulling may not alter specific fatty acids in faba beans in the same way. After dehulling, all fatty acids except oleic acid decreased in Brocal, Pirkkonen, and Strumicka, whereas Seville decreased in all but linoleic acid. Despite these variances, dehulling had no significant effect on any of the fatty acid levels, causing only a slight increase or decrease in specific cultivars (Table 1) as well as the whole population (Figure S1). Dehulling is believed to promote lipid degradation and, hence, expose fatty acids in legumes [47]. Despite this claim, only a few studies attempted to investigate the effect of dehulling on individual fatty acid contents in legumes [1,48]. As far as we can tell, this is the first study to show the effect of dehulling on individual fatty acids in faba bean cultivars. Our study demonstrated that dehulling had no significant effect on the levels of fatty acids in faba beans. Therefore, an additional dehulling procedure might not add significant value during fatty acid extraction from faba beans.

### 3.4. TPC, TTC, and TSC

The levels of TPC, TTC, and TSC in the whole seeds and dehulled seeds of the 22 faba bean cultivars are presented in Table 2, and significant variations were observed (*p* < 0.05). TPC in the whole seeds ranged from 2.04 mg GAE/g in NPL-JSW-2003-65 to 6.11 mg GAE/g in Seville, showing an approximately 3-fold difference (*p* < 0.05). In the dehulled seeds, TPC ranged from 2.86 mg GAE/g in Domasna-2 to 4.77 mg GAE/g in Tempranas De Machamiel (*p* < 0.05). Likewise, TSC and TTC were in the ranges of 2.94–7.84 mg CE/g and 5.81–9.71 mg DE/g in the whole seeds and 1.85–3.50 mg CE/g and 5.78–8.81 mg DE/g in the dehulled seeds, respectively. Among the faba bean cultivars, Large Mazandaran and Muchamiel displayed the highest TTC while Giant Three Seeded and Levens Marschbohne displayed the lowest in their whole seeds and dehulled seeds, respectively. In terms of TSC, Zborovicki in its whole seeds and Dosmasna-1 in its dehulled seeds displayed the highest contents, while MMR-KJT-2010-K161716 and Pirkkonen displayed the lowest contents, respectively. The TPC, TTC, and TSC levels observed in whole seeds are comparable to numerous prior findings. Karats et al. [32], for example, discovered a TPC of 2.9 mg GAE/g and a TTC of 1.9 mg CE/g in a faba bean variety from Turkey, with the former equivalent to several of the cultivars in our study and the latter being much lower. In another investigation, Oomah et al. [49] discovered TPC ranging from 5.59 to 40.63 mg CE/g and TTC ranging from 0.50 to 5.23 mg CE/g across thirteen faba bean genotypes grown in Canada. Other studies also discovered a wide range of TTC, TPC, and TSC levels, which could be ascribed to differences in genotype, extraction methodology, reporting method, and post-harvest handling [37,50,51].

The effect of dehulling on TPC, TSC, and TTC levels in each cultivar as well as the whole population was statistically evaluated (Table 2, Figure S1). Dehulling decreased the TPC level in five cultivars including Pirkkonen, Seville, Large Mazandaran, Aguadulce, and Domasna-2, with the observed decrease in the first two cultivars being significant (*p* < 0.01). In the remaining cultivars, dehulling increased the TPC level with thirteen cultivars exhibiting a significant rise. The highest increase was observed in Zborovicki (49.58%) while the lowest was in Strumicka (1.42%). Rajhi et al. [22] recently investigated the influence of dehulling on TPC in two Tunisian faba bean cultivars, discovering an increase in TPC in one cultivar and a decrease in the other, albeit the differences were insignificant. The distribution of phenolic compounds in legume seed sections varies greatly, with some having a high concentration in their seed coats and others in their cotyledon [16]. As a result, the effect of dehulling varies greatly amongst legumes as well as genotypes of the same species. Overall, our study discovered that dehulling may not have a consistent influence on TPC levels in faba bean cultivars. As a result, the suitability of individual genotypes should be scrutinized (Table 2). TSC also followed a similar pattern to TPC in that dehulling enhanced the level of TSC in most of the cultivars but decreased its level in seven cultivars. However, the observed rise in TSC was not significant, except for Ascott, which showed the largest increase (8.61%) (*p* < 0.05). Among the seven cultivars that showed a drop in TSC after dehulling, four (Zborovicki, Pirkkonen, Large Mazandaran, and Abawi# 1) showed a significant decrease. Unlike the TPC and TSC, dehulling decreased the level of TTC in all the faba bean cultivars, with all reductions except for Brocal being significant. In support of our findings, several previous studies have shown a significant reduction in TTC levels in faba beans following dehulling [2,9,15,19,20]. Other studies also indicated comparable results in legumes other than faba beans, such as lentils, chickpeas, mung beans, and cowpeas [35,48,52]. Tannins, which are abundant in seed coats, are anti-nutrient factors that alter the digestibility and bioavailability of proteins and minerals [24]. As a result, dehulling faba beans, like in other legumes, could be a useful processing method for reducing the anti-nutrient effect of tannins. On the other hand, there are also benefits to consuming faba beans with high levels of tannins. One advantage is that feeding tannin-enriched faba beans, in combination with flaxseed, to dairy cows has the potential to improve their milk production and increase the amount of beneficial fatty acids found in the milk [53]. Hence, it is important to acknowledge that feeding dehulled faba beans may have negative impacts on these and related aspects.

### 3.5. DPPH^•^-Scavenging Activity, ABTS^•+^-Scavenging Activity, and FRAP

The antioxidant activities of the whole and dehulled seeds of the faba bean cultivars were assessed using three distinct assays, including FRAP, DPPH^•^-scavenging activity, and ABTS^•+^-scavenging activity. As shown in Table 3, significant variations were observed between the faba bean cultivars (*p* < 0.05). FRAP and DPPH^•^-scavenging activity were in the ranges of 0.69–3.62 and 0.35–2.24 mg AAE/g in the whole seeds and 0.89–2.69 and 0.53–2.45 mg AAE/g in the dehulled seeds, respectively. Likewise, ABTS^•+^-scavenging activity ranged from 3.07 to 7.34 mg TE/g in the whole seeds and from 4.55 to 10.34 mg TE/g in the dehulled seeds. In the whole seeds, Pirkkonen had the highest FRAP and ABTS^•+^-scavenging activity but the lowest DPPH^•^-scavenging activity. This cultivar had the second-highest TPC and TTC, as well as the fourth-highest TSC. Seville, which had the highest TPC, displayed the highest DPPH^•^-scavenging activity. In the dehulled seeds, Muchamiel had the highest FRAP, while Primus showed the highest ABTS^•+^-scavenging activity and DPPH^•^-scavenging activity. MMR-KJT-2010-K161716 showed the lowest FRAP, Pirkkonen the lowest ABTS^•+^-scavenging activity, and Domasna-2 the lowest DPPH^•^-scavenging activity. In support of our findings, previous studies also showed the antioxidant activities of various faba bean genotypes. For example, DPPH^•^-scavenging activity and FRAP in the ranges of 2.7–7.8 and 10.92–20.40 mol TE/g were found in four faba beans grown in Australia [54]. Furthermore, a DPPH^•^-scavenging activity ranging from 13.75 to 108.00 µg/mL and a total antioxidant activity ranging from 6.44 to 7.89 mg GAE/g were observed in different sample types of two Tunisian faba bean cultivars [22]. Similarly, FRAP ranged from 0.696 to 4.461 mmol/g and DPPH^•^-scavenging activity ranged from 14.99 to 78.13% in mature seeds of fourteen faba beans [55]. As demonstrated in these studies, discrepancies in assays, extraction techniques, and reporting methods make comparison challenging. Overall, our results demonstrated a wide range of antioxidant activity in the faba bean cultivars. It should be noted that many of the cultivars with high levels of TPC, TTC, and TSC showed higher antioxidant activity, demonstrating the role of such metabolites in regulating reactive radicals [27].

The effect of dehulling on each of the antioxidant activities was also analyzed (Table 3, Figure S1), and the results are comparable to those of TPC and TSC results. While two cultivars, Domasna-2 and Yavneh, showed a decrease in all antioxidant activities, five cultivars, Ascott, Giant Three Seeded, Levens Marschbohne, Primus, and Zborovicki, showed an increase. The remaining cultivars had varying antioxidant activity results, with one increasing and the other decreasing or vice versa. Pirkonnen showed the highest declines in ABTS^•+^-scavenging activity (37.94%) and FRAP (59.69%) and the highest rise in DPPH^•^-scavenging activity (73.61%). On the other hand, Zborovicki showed the highest increases in ABTS^•+^-scavenging activity (63.93%) and FRAP (48.26%). The highest drop in DPPH^•^-scavenging activity (49.46%) was observed in Domasna-2. The observed increases and/or decreases in the antioxidant activities after dehulling were significantly different in all the faba bean cultivars except for Muchamiel, Brocal, Algerian, and Abawi# 1. Studies on the effect of dehulling on antioxidant activity in legumes, particularly faba beans, are limited. Previously, two separate studies on the effect of dehulling on antioxidant activity in faba beans reported conflicting results. Dehulling decreased DPPH^•^-scavenging activity up to 33.33%, while showing an inverse effect on iron-reducing power [22]. In Chaieb et al.’s [55] study, dehulling reduced both FRAP and DPPH^•^-scavenging activity. Overall, our study indicated that dehulling may not equally affect faba bean genotypes in terms of their antioxidant activity. This could be due to the variable distribution of molecules responsible for antioxidant activity among different faba bean genotypes [16,17]. Therefore, individual genotypes should be evaluated for their suitability for dehulling processes.

### 3.6. Principal Component (PCA) and Correlation Analyses

**Figure 3:**
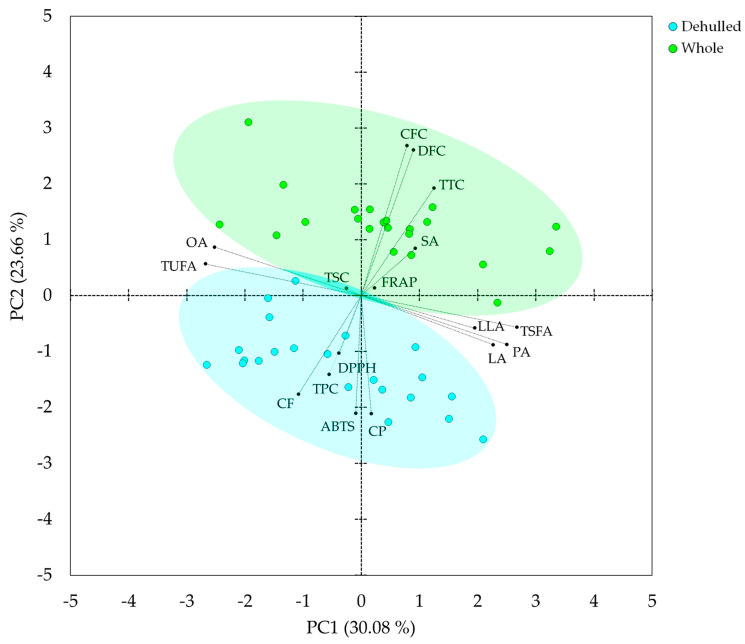
Bi-plot of variables and observations obtained from principal component analysis. ABTS: ABTS^•+^-scavenging activity; CFC: Crude fiber content; DFC: Dietary fiber content; DPPH: DPPH^•^-scavenging activity; FRAP: Ferric-reducing antioxidant power; LA: Linoleic acid; LLA: Linolenic acid; OA: Oleic acid; PA: Palmitic acid; SA: Stearic acid; CF: Crude fat; CP: Crude protein; TPC: Total phenolic content; TSC: Total saponin content; TSFA: Total saturated fatty acid; TTC: Total tannin content; TUFA: Total unsaturated fatty acid.

PCA was computed to further view how the faba bean cultivars responded to dehulling and see their association with the nutritional and secondary metabolite components. The PCA yielded four components with eigenvalues of greater than 1 which explained a cumulative variance of 77.46%. As shown in the bi-plot (Figure 3), the dehulled seeds and whole seeds were clearly separated along the first two principal components (PC1 and PC2), which together explained more than 50% of the total variance. The dehulled seeds were distributed along the positive end of PC1 and the negative end of PC2, while whole seeds were distributed along the positive ends of both PC1 and PC2. As shown in Table 4 and the bi-plot (Figure 3), palmitic acid, linoleic acid, linolenic acid, and TSFA were the major contributors along PC1, having positive FL above 0.5, while only oleic acid and TUFA having negative FL above 0.5. Along PC2, variables with positive FL above 0.5 were TTC, CFC, and DFC, and variables with a negative FL above 0.5 were ABTS^•+^-scavenging activity, total protein, and total fat. Overall, the distinct separation of dehulled seeds and whole seeds in the PCA demonstrates their variations in nutritional and functional metabolite distribution, as well as antioxidant activity.

**Figure 4:**
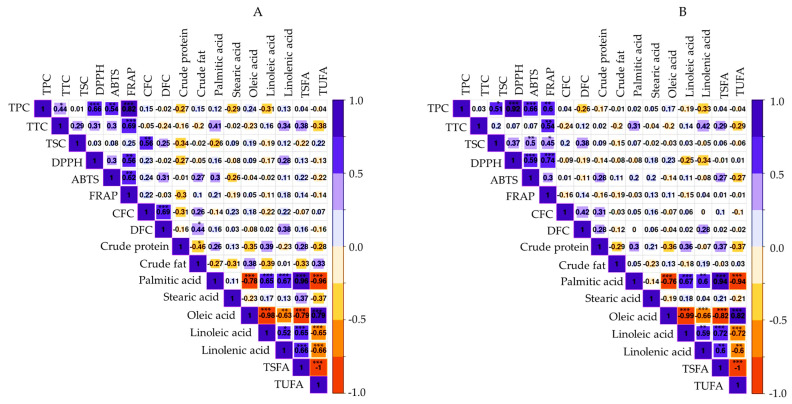
Correlation matrix of variable in the whole seeds (**A**) and dehulled seeds (**B**). ABTS: ABTS^•+^-scavenging activity; CFC: Crude fiber content; DFC: Dietary fiber content; DPPH: DPPH^•^-scavenging activity; FRAP: Ferric-reducing antioxidant power; TPC: Total phenolic content; TSC: Total saponin content; TSFA: Total saturated fatty acid; TTC: Total tannin content; TUFA: Total unsaturated fatty acid. * *p* < 0.05, ** *p* < 0.01, *** *p* < 0.001.

Correlation analysis was also performed to examine the relationship between the parameters studied utilizing the entire data set in each sample type (Figure 4A,B). TPC showed strong and positive correlations with each of the antioxidant activities (*r* ≥ 0.54) at different levels of significance regardless of sample type. These observations corroborate many previous studies and signify the role of phenolic components in both the whole seeds and dehulled seeds in controlling reactive radicals [54,55]. The antioxidant activities also showed positive correlations with each other. The relationship between CFC and DFC was significant in whole seeds (*r* = 0.69, *p* < 0.001) which could be attributed to their abundance compared to the dehulled seeds. Oleic acid showed negative correlations with the other unsaturated fatty acids in both whole seeds and dehulled seeds. Other notable negative correlations such as total protein with total fat, TPC, and TSC could be attributed to their distinct biosynthesis pathways.

## 4. Conclusions

This study investigated the effect of dehulling on nutrient levels, functional metabolite contents, and antioxidant activities in faba bean cultivars that are widely grown in different countries. Significant variations in the analyzed metabolites and antioxidant activities were observed between the faba bean cultivars. The faba bean cultivars that simultaneously displayed high nutrient levels, functional metabolite contents, and antioxidant activities could therefore be ideal resources. Dehulling the faba beans also affected the levels of nutrients, functional metabolites, and antioxidant activities, but in different ways. While dehulling reduced the amount of dietary fiber, crude fiber, and total tannin, the process increased the contents of crude protein and crude fat. Concerning other parameters, including fatty acids, total phenol, total saponin, and antioxidant activities, dehulling showed variable results. Therefore, individual genotypes should be assessed for their suitability for the process. In general, dehulling could be an ideal processing procedure for increasing protein and fat contents in faba beans while decreasing tannin concentration, one of the anti-nutrient factors. However, the process costs the high fiber level to be obtained from consuming whole beans. Generally, the findings of this study could provide a detailed understanding of how dehulling affects the different metabolites of faba beans. Moreover, the study brings attention to the variations in metabolites and antioxidant activities among different faba bean genotypes, indicating genetic distinctions. This could potentially pave the way for future molecular-level examinations. However, due to limited access to a diverse range of genotypes, the study was unable to statistically analyze the impact of origin on the analyzed parameters. Therefore, it is strongly recommended that future studies focus on this aspect. Additionally, the findings from this research could serve as a foundation for further exploration of other anti-nutrient factors found in various faba bean genotypes.

## Figures and Tables

**Table 1:** Variations of fatty acid contents in whole and dehulled seeds of 22 faba bean cultivars.

Cultivar	Sample	Fatty Acid Contents (%)						
		Palmitic Acid	Stearic Acid	Oleic Acid	Linoleic Acid	Linolenic Acid	TSFA	TUFA
Abawi# 1	Whole	16.33 ± 0.02 ^j^	2.58 ± 0.01 ^g^	29.93 ± 0.05 ^l^	48.74 ± 0.04 ^e^	2.43 ± 0.02 ^d^	18.91 ± 0.01 ^l^	81.09 ± 0.01 ^e^
	Dehulled	16.29 ± 0.05 ^l^ (−0.23) ^ns^	2.37 ± 0.01 ^h^ (−8.29) ^ns^	28.23 ± 0.07 ^l^ (−5.69) ^ns^	50.42 ± 0.08 ^f^ (3.34) ^ns^	2.69 ± 0.04 ^c^ (9.94) ^ns^	18.66 ± 0.05 ^l^ (−1.33) ^ns^	81.34 ± 0.05 ^d^ (0.31) ^ns^
Aguadulce	Whole	18.57 ± 0.05 ^b^	2.41 ± 0.01 ^jk^	25.53 ± 0.10 °	50.82 ± 0.10 ^d^	2.67 ± 0.05 ^bc^	20.98 ± 0.05 ^c^	79.02 ± 0.05 ^n^
	Dehulled	18.57 ± 0.01 ^b^ (−0.04) ^ns^	2.13 ± 0.00 ° (−11.47) ^ns^	25.62 ± 0.07 ^p^ (0.36) ^ns^	51.02 ± 0.06 ^e^ (0.41) ^ns^	2.65 ± 0.01 ^c^ (−0.59) ^ns^	20.70 ± 0.01 ^b^ (−1.35) ^ns^	79.30 ± 0.01 ^n^ (0.36) ^ns^
Algerian	Whole	16.83 ± 0.06 ^h^	2.57 ± 0.01 ^g^	30.78 ± 0.13 ^j^	47.77 ± 0.16 ^g^	2.05 ± 0.03 ^h^	19.40 ± 0.07 ^f^	80.60 ± 0.07 ^k^
	Dehulled	16.95 ± 0.04 ^i^ (0.69) ^ns^	2.20 ± 0.01 ^m^ (−14.67) ^ns^	31.43 ± 0.02 ^i^ (2.08) ^ns^	47.23 ± 0.20 ^j^ (−1.14) ^ns^	2.20 ± 0.07 ^g^ (6.92) ^ns^	19.14 ± 0.05 ^j^ (−1.34) ^ns^	80.86 ± 0.05 ^f^ (0.32) ^ns^
Alicante	Wholef	17.21 ± 0.00 ^f^	2.75 ± 0.01 ^e^	31.74 ± 0.07 ^i^	46.28 ± 0.07 ^i^	2.01 ± 0.02 ^h^	19.96 ± 0.02 ^f^	80.04 ± 0.02 ^k^
	Dehulled	17.29 ± 0.03 ^g^ (0.43) ^ns^	2.47 ± 0.02 ^g^ (−10.10) ^ns^	30.61 ± 0.05 ^j^ (−3.54) ^ns^	47.62 ± 0.06 ^i^ (2.81) ^ns^	2.00 ± 0.02 ^ij^ (−0.50) ^ns^	19.76 ± 0.03 ^g^ (−1.02) ^ns^	80.24 ± 0.03 ^i^ (0.25) ^ns^
Ascott	Whole	15.87 ± 0.04 ^l^	2.22 ± 0.01 ^n^	34.83 ± 0.11 ^d^	45.05 ± 0.12 ^k^	2.03 ± 0.02 ^h^	18.09 ± 0.03 °	81.91 ± 0.03 ^b^
	Dehulled	16.52 ± 0.03 ^k^ (3.91) ^ns^	1.86 ± 0.01 ^r^ (−16.19) ^ns^	34.37 ± 0.02 ^e^ (−1.31) ^ns^	45.20 ± 0.12 ^m^ (0.33) ^ns^	2.05 ± 0.00 ^hi^ (1.06) ^ns^	18.38 ± 0.03 ^m^ (1.56) ^ns^	81.62 ± 0.03 ^c^ (−0.35) ^ns^
Brocal	Whole	17.50 ± 0.03 ^e^	3.14 ± 0.00 ^b^	32.16 ± 0.09 ^h^	44.65 ± 0.09 ^l^	2.55 ± 0.02 ^c^	20.64 ± 0.03 ^d^	79.36 ± 0.03 ^m^
	Dehulled	16.17 ± 0.03 ^m^ (−7.59) ^ns^	2.69 ± 0.01 ^d^ (−14.26) ^ns^	36.42 ± 0.00 ^b^ (11.70) ^ns^	42.25 ± 0.11 ^q^ (−5.37) ^ns^	2.47 ± 0.02 ^ef^ (−3.29) ^ns^	18.86 ± 0.03 ^k^ (−8.61) ^ns^	81.14 ± 0.03 ^e^ (2.19) ^ns^
Domasna-1	Whole	16.80 ± 0.02 ^h^	2.90 ± 0.01 ^c^	29.73 ± 0.03 ^l^	48.22 ± 0.04 ^f^	2.35 ± 0.00 ^de^	19.70 ± 0.01 ^gh^	80.30 ± 0.01 ^ij^
	Dehulled	16.70 ± 0.02 ^j^ (−7.37) ^ns^	2.91 ± 0.00 ^b^ (−9.15) ^ns^	27.05 ± 0.05 ^m^ (8.38) ^ns^	50.91 ± 0.04 ^e^ (−3.49) ^ns^	2.43 ± 0.01 ^f^ (8.47) ^ns^	19.61 ± 0.03 ^h^ (−7.60) ^ns^	80.39 ± 0.03 ^h^ (1.83) ^ns^
Domasna-2	Whole	17.29 ± 0.01 ^f^	2.44 ± 0.01 ^ij^	32.50 ± 0.05 ^g^	45.71 ± 0.05 ^j^	2.05 ± 0.01 ^h^	19.74 ± 0.02 ^g^	80.26 ± 0.02 ^j^
	Dehulled	16.02 ± 0.08 ^n^ (−0.63) ^ns^	2.22 ± 0.00 ^l^ (0.58) ^ns^	35.47 ± 0.26 ^c^ (−9.02) ^ns^	44.05 ± 0.29 ^n^ (5.28) ^ns^	2.24 ± 0.05 ^g^ (3.40) ^ns^	18.24 ± 0.08 ^n^ (−0.45) ^ns^	81.76 ± 0.08 ^b^ (0.11) ^ns^
Ethiopia 530	Whole	16.14 ± 0.06 ^k^	2.22 ± 0.01 ^n^	33.69 ± 0.22 ^f^	45.61 ± 0.25 ^j^	2.33 ± 0.04 ^de^	18.37 ± 0.06 ^n^	81.63 ± 0.06 ^c^
	Dehulled	15.83 ± 0.04 ° (−1.94) ^ns^	2.17 ± 0.00 ^n^ (−2.40) ^ns^	33.78 ± 0.11 ^g^ (0.28) ^ns^	46.14 ± 0.13 ^k^ (1.14) ^ns^	2.08 ± 0.02 ^h^ (−10.74) ^ns^	18.00 ± 0.04 ° (−2.00) ^ns^	82.00 ± 0.04 ^e^ (0.45) ^ns^
Giant Three Seeded	Whole	15.38 ± 0.02 ^m^	2.44 ± 0.00 ^ij^	39.13 ± 0.07 ^b^	41.18 ± 0.05 °	1.87 ± 0.01 ^i^	17.83 ± 0.02 ^p^	82.17 ± 0.02 ^a^
	Dehulled	16.02 ± 0.05 ^n^ (4.00) ^ns^	2.23 ± 0.01 ^l^ (−8.70) ^ns^	34.10 ± 0.15 ^f^ (−12.85) ^ns^	45.82 ± 0.13 ^l^ (10.14) ^ns^	1.83 ± 0.02 ^k^ (−2.49) ^ns^	18.25 ± 0.06 ^n^ (2.35) ^ns^	81.75 ± 0.06 ^b^ (−0.52) ^ns^
Large Mazandaran	Whole	18.83 ± 0.16 ^a^	2.44 ± 0.01 ^ij^	24.32 ± 0.19 ^p^	51.30 ± 0.27 ^c^	3.11 ± 0.07 ^a^	21.27 ± 0.17 ^b^	78.73 ± 0.17 °
	Dehulled	19.09 ± 0.01 ^a^ (1.37) ^ns^	2.06 ± 0.00 ^q^ (−15.49) ^ns^	24.14 ± 0.05 ^r^ (−0.73) ^ns^	51.57 ± 0.04 ^c^ (0.53) ^ns^	3.13 ± 0.02 ^a^ (0.70) ^ns^	21.15 ± 0.02 ^a^ (−0.54) ^ns^	78.85 ± 0.02 ° (0.15) ^ns^
Levens Marschbohne	Whole	16.50 ± 0.11 ^i^	2.79 ± 0.02 ^d^	23.87 ± 0.12 ^q^	54.44 ± 0.24 ^a^	2.41 ± 0.00 ^d^	19.29 ± 0.13 ^jk^	80.71 ± 0.13 ^fg^
	Dehulled	17.19 ± 0.08 ^h^ (3.98) ^ns^	2.64 ± 0.01 ^e^ (−5.24) ^ns^	22.43 ± 0.09 ^s^ (−6.02) ^ns^	55.28 ± 0.13 ^a^ (1.52) ^ns^	2.46 ± 0.04 ^ef^ (2.33) ^ns^	19.83 ± 0.09 ^fg^ (2.71) ^ns^	80.17 ± 0.09 ^ij^ (−0.67) ^ns^
MMR-KJT-2010-K161716	Whole	17.57 ± 0.05 ^e^	2.32 ± 0.01 ^m^	29.34 ± 0.16 ^m^	48.77 ± 0.10 ^e^	2.00 ± 0.03 ^h^	19.89 ± 0.05 ^f^	80.11 ± 0.05 ^k^
	Dehulled	17.79 ± 0.06 ^d^ (1.21) ^ns^	2.09 ± 0.00 ^p^ (−9.98) ^ns^	28.16 ± 0.09 ^l^ (−4.02) ^ns^	49.77 ± 0.11 ^g^ (2.02) ^ns^	2.19 ± 0.04 ^g^ (8.87) ^ns^	19.88 ± 0.07 ^f^ (−0.09) ^ns^	80.12 ± 0.07 ^j^ (0.02) ^ns^
Muchamiel	Whole	18.09 ± 0.08 ^d^	2.38 ± 0.00 ^kl^	25.49 ± 0.21 °	51.27 ± 0.22 ^c^	2.77 ± 0.07 ^b^	20.47 ± 0.08 ^e^	79.53 ± 0.08 ^l^
	Dehulled	18.01 ± 0.02 ^c^ (−0.46) ^ns^	2.28 ± 0.00 ^k^ (−4.26) ^ns^	24.57 ± 0.03 ^q^ (−3.60) ^ns^	52.17 ± 0.01 ^b^ (1.73) ^ns^	2.98 ± 0.01 ^b^ (6.83) ^ns^	20.28 ± 0.02 ^d^ (−0.91) ^ns^	79.72 ± 0.02 ^l^ (0.23) ^ns^
NPL-JSW-2003-65	Whole	18.25 ± 0.04 ^c^	3.26 ± 0.02 ^a^	22.97 ± 0.14 ^r^	52.93 ± 0.07 ^b^	2.59 ± 0.01 ^c^	21.50 ± 0.06 ^a^	78.50 ± 0.06 ^p^
	Dehulled	16.95 ± 0.01 ^i^ (−7.14) ^ns^	3.26 ± 0.00 ^a^ (0.21) ^ns^	26.69 ± 0.09 ^n^ (13.93) ^ns^	50.51 ± 0.09 ^f^ (−4.58) ^ns^	2.59 ± 0.01 ^d^ (−0.06) ^ns^	20.21 ± 0.02 ^d^ (−6.03) ^ns^	79.79 ± 0.01 ^l^ (1.62) ^ns^
Pirkkonen	Whole	17.07 ± 0.02 ^g^	2.47 ± 0.00 ^i^	31.55 ± 0.07 ^i^	46.84 ± 0.04 ^h^	2.08 ± 0.01 ^h^	19.53 ± 0.02 ^hi^	80.47 ± 0.02 ^hi^
	Dehulled	16.03 ± 0.04 ^n^ (−6.06) ^ns^	2.30 ± 0.01 ^j^ (−6.87) ^ns^	33.62 ± 0.03 ^g^ (6.18) ^ns^	46.08 ± 0.15 ^k^ (−1.63) ^ns^	1.97 ± 0.02 ^j^ (−5.32) ^ns^	18.33 ± 0.04 ^mn^ (6.16) ^ns^	81.67 ± 0.04 ^bc^ (1.47) ^ns^
Primus	Whole	16.83 ± 0.16 ^h^	2.73 ± 0.04 ^e^	30.43 ± 0.16 ^k^	48.20 ± 0.28 ^f^	1.80 ± 0.05 ^i^	19.56 ± 0.17 ^hi^	80.44 ± 0.17 ^hi^
	Dehulled	17.62 ± 0.03 ^e^ (4.50) ^ns^	2.79 ± 0.01 ^c^ (2.09) ^ns^	29.08 ± 0.08 ^k^ (−4.44) ^ns^	48.70 ± 0.07 ^h^ (1.01) ^ns^	1.81 ± 0.02 ^k^ (0.37) ^ns^	20.41 ± 0.04 ^c^ (4.17) ^ns^	79.59 ± 0.04 ^m^ (−1.06) ^ns^
Seville	Whole	16.38 ± 0.02 ^ij^	2.34 ± 0.01 l^m^	39.54 ± 0.05 ^a^	39.53 ± 0.06 ^p^	2.21 ± 0.03 ^fg^	18.72 ± 0.03 ^m^	81.28 ± 0.03 ^d^
	Dehulled	16.14 ± 0.01 ^m^ (−1.42) ^ns^	2.23 ± 0.00 ^l^ (−4.91) ^ns^	39.50 ± 0.04 ^a^ (−0.11) ^ns^	40.09 ± 0.05 ^r^ (1.39) ^ns^	2.05 ± 0.01 ^hi^ (−7.40) ^ns^	18.37 ± 0.01 ^m^ (−1.86) ^ns^	81.63 ± 0.01 ^c^ (0.43) ^ns^
Strumicka	Whole	16.72 ± 0.07 ^h^	2.52 ± 0.04 ^h^	32.35 ± 0.14 ^gh^	46.30 ± 0.12 ^i^	2.11 ± 0.03 ^gh^	19.24 ± 0.04 ^k^	80.76 ± 0.04 ^f^
	Dehulled	16.30 ± 0.02 ^l^ (−2.48) ^ns^	2.35 ± 0.02 ^hi^ (−6.75) ^ns^	33.16 ± 0.09 ^h^ (2.43) ^ns^	46.12 ± 0.09 ^k^ (−0.40) ^ns^	2.07 ± 0.02 ^h^ (−1.65) ^ns^	18.65 ± 0.04 ^l^ (−3.04) ^ns^	81.35 ± 0.04 ^d^ (0.72) ^ns^
Tempranas De Machamiel	Whole	16.84 ± 0.01 ^h^	2.67 ± 0.00 ^f^	34.25 ± 0.03 ^e^	43.88 ± 0.04 ^m^	2.35 ± 0.00 ^de^	19.51 ± 0.01 ^i^	80.49 ± 0.01 ^h^
	Dehulled	16.90 ± 0.02 ^i^ (0.36) ^ns^	2.34 ± 0.01 ^i^ (−12.29) ^ns^	34.77 ± 0.06 ^d^ (1.49) ^ns^	43.79 ± 0.05 ° (−0.22) ^ns^	2.20 ± 0.01 ^g^ (−6.55) ^ns^	19.25 ± 0.01 ^i^ (−1.37) ^ns^	80.75 ± 0.01 ^g^ (0.33) ^ns^
Yavneh	Whole	17.22 ± 0.10 ^f^	2.35 ± 0.04 ^lm^	27.41 ± 0.45 ^n^	50.76 ± 0.10 ^d^	2.26 ± 0.21 ^ef^	19.57 ± 0.14 ^hi^	80.43 ± 0.14 ^hi^
	Dehulled	17.46 ± 0.06 ^f^ (1.37) ^ns^	2.59 ± 0.00 ^f^ (9.27) ^ns^	26.15 ± 0.03 ° (−4.60) ^ns^	51.31 ± 0.08 ^d^ (1.06) ^ns^	2.49 ± 0.01 ^e^ (9.37) ^ns^	20.05 ± 0.06 ^e^ (2.39) ^ns^	79.95 ± 0.06 ^k^ (−0.60) ^ns^
Zborovicki	Whole	14.93 ± 0.10 ^n^	2.79 ± 0.01 ^d^	38.79 ± 0.11 ^c^	41.47 ± 0.17 ^n^	2.02 ± 0.02 ^h^	17.72 ± 0.09 ^p^	82.28 ± 0.09 ^a^
	Dehulled	15.51 ± 0.03 ^p^ (3.73) ^ns^	2.78 ± 0.01 ^c^ (−0.39) ^ns^	36.54 ± 0.03 ^b^ (−5.79) ^ns^	43.09 ± 0.04 ^p^ (3.76) ^ns^	2.08 ± 0.01 ^h^ (2.76) ^ns^	18.29 ± 0.03 ^mn^ (3.11) ^ns^	81.71 ± 0.03 ^bc^ (0.69) ^ns^
Total range	Whole	4.93–18.83	2.22–3.26	22.97–39.54	39.53–54.44	1.80–3.11	17.72–21.50	78.50–82.28
	Dehulled	15.51–19.09	1.86–3.26	22.43–39.50	40.09–55.28	1.81–3.13	18.00–21.15	78.85–82.00
Total mean	Whole	16.96	2.58	30.92	47.26	2.27	19.54	80.46
	Dehulled	16.88	2.41	30.72	47.69	2.30	19.29	80.71
CV (%)	Whole	5.55	10.43	15.06	7.92	13.80	5.16	1.25
	Dehulled	5.37	13.37	15.10	7.78	14.95	4.76	1.14

Different superscript letters in a column show significantly different mean values between the faba bean cultivars at *p* < 0.05 for the same sample type. Values in parentheses indicate % increase (positive values) or % decrease (negative sign) after dehulling. ^ns^ Not significant at *p* < 0.05. TSFA: Total saturated fatty acid, TUFA: Total unsaturated fatty acid.

**Table 2:** Variations of total phenolic content, total saponin content, and total tannin content in whole and dehulled seeds of 22 faba bean cultivars.

Cultivar	Total Phenolic Content (mg GAE/g)		Total Saponin Content (mg DE/g)		Total Tannin Content (mg CE/g)	
	Whole	Dehulled	Whole	Dehulled	Whole	Dehulled
Abawi# 1	2.85 ± 0.08 ^ghi^	3.39 ± 0.07 ^f^ (16.12) **	9.55 ± 0.32 ^ab^	8.02 ± 0.69 ^a–d^ (−16.09) *	4.40 ± 0.20 ^d–g^	3.45 ± 0.04 ^a^ (−21.57) **
Aguadulce	3.81 ± 0.12 ^cd^	3.54 ± 0.10 ^ef^ (−6.85) ^ns^	6.26 ± 0.83 ^ij^	7.36 ± 0.44 ^a–d^ (14.98) ^ns^	4.59 ± 0.11 ^c–f^	2.95 ± 0.32 ^bc^ (−35.78) **
Algerian	2.76 ± 0.07 ^hi^	3.07 ± 0.11 ^g^ (10.02) *	8.46 ± 0.62 ^b–e^	8.12 ± 0.35 ^a–d^ (−4.02) ^ns^	3.19 ± 0.27 ^ij^	2.37 ± 0.14 ^fgh^ (−25.55) *
Alicante	3.54 ± 0.23 ^de^	3.95 ± 0.18 ^bcd^ (10.35) ^ns^	7.57 ± 0.84 ^d–h^	7.63 ± 0.32 ^a–d^ (0.71) ^ns^	5.11 ± 0.49 ^bcd^	3.11 ± 0.17 ^b^ (−39.06) **
Ascott	2.14 ± 0.12 ^j^	3.94 ± 0.01 ^bcd^ (45.63) ***	7.93 ± 0.21 ^c–g^	8.67 ± 0.31 ^ab^ (8.61) *	3.44 ± 0.07 ^hij^	2.07 ± 0.04 ^hi^ (−39.90) ***
Brocal	2.64 ± 0.23 ^i^	3.31 ± 0.14 ^f^ (20.24) *	8.25 ± 0.18 ^c–f^	7.75 ± 0.71 ^a–d^ (−6.08) ^ns^	3.01 ± 0.40 ^ij^	2.96 ± 0.14 ^bc^ (−1.70) ^ns^
Domasna-2	2.96 ± 0.11 ^ghi^	2.86 ± 0.07 ^g^ (−3.57) ^ns^	7.09 ± 0.74 ^f–i^	6.57 ± 0.50 ^de^ (−7.31) ^ns^	4.18 ± 0.14 ^e–h^	2.49 ± 0.11 ^d–g^ (−40.44) **
Domasna-1	3.54 ± 0.07 ^de^	4.05 ± 0.07 ^bc^ (12.61) **	7.30 ± 0.81 ^e–i^	8.81 ± 0.74 ^a^ (17.19) ^ns^	5.44 ± 0.41 ^bc^	2.63 ± 0.04 ^c–f^ (−51.75) **
Ethiopia 530	3.14 ± 0.08 ^fg^	3.95 ± 0.05 ^bcd^ (20.51) **	7.30 ± 0.48 ^e–i^	8.20 ± 0.23 ^abc^ (10.94) ^ns^	3.62 ± 0.24 ^g–j^	2.63 ± 0.17 ^c–f^ (−27.44) **
Giant Three Seeded	3.20 ± 0.17 ^efg^	3.99 ± 0.19 ^bcd^ (19.83) *	6.64 ± 0.26 ^g–j^	7.32 ± 0.70 ^a–d^ (9.29) ^ns^	2.94 ± 0.09 ^j^	1.93 ± 0.21 ^i^ (−34.19) **
Large Mazandaran	3.88 ± 0.16 ^c^	3.74 ± 0.08 ^de^ (−3.62) ^ns^	9.00 ± 0.87 ^a–c^	7.19 ± 0.22 ^b–e^ (−20.16) *	7.84 ± 0.52 ^a^	2.76 ± 0.08 ^b–e^ (−64.78) **
Levens Marschbohne	2.71 ± 0.18 ^i^	4.01 ± 0.12 ^bcd^ (32.48) **	7.17 ± 0.77 ^e–i^	7.86 ± 0.19 ^a–d^ (8.72) ^ns^	3.79 ± 0.56 ^f–j^	1.85 ± 0.13 ^i^ (−51.27) **
MMR-KJT-2010-K161716	2.31 ± 0.14 ^j^	3.77 ± 0.21 ^cde^ (38.91) **	5.81 ± 0.27 ^j^	7.35 ± 0.81 ^a–d^ (21.03) ^ns^	5.23 ± 0.48 ^bcd^	2.47 ± 0.14 ^efg^ (−52.88) **
Muchamiel	3.45 ± 0.21 ^def^	3.83 ± 0.21 ^cd^ (9.92) ^ns^	7.73 ± 0.30 ^d–g^	7.99 ± 0.97 ^a–d^ (3.33) ^ns^	4.87 ± 0.07 ^cde^	3.50 ± 0.27 ^a^ (−28.09) **
NPL-JSW-2003-65	2.04 ± 0.01 ^j^	3.36 ± 0.15 ^f^ (39.34) **	6.29 ± 0.38 ^h–j^	6.74 ± 0.12 ^cde^ (6.63) ^ns^	4.61 ± 0.17 ^c–f^	1.96 ± 0.14 ^i^ (−57.59) ***
Pirkkonen	4.53 ± 0.10 ^b^	3.42 ± 0.08 ^f^ (−24.67) **	8.78 ± 0.43 ^a–d^	5.78 ± 0.14 ^e^ (−34.17) **	7.08 ± 0.93 ^a^	2.34 ± 0.14 ^fgh^ (−66.99) **
Primus	3.11 ± 0.29 ^fgh^	4.76 ± 0.09 ^a^ (34.81) **	7.59 ± 0.78 ^d–h^	8.64 ± 0.65 ^ab^ (12.15) ^ns^	4.97 ± 0.43 ^b–e^	2.87 ± 0.27 ^bc^ (−42.29) **
Seville	6.11 ± 0.15 ^a^	4.63 ± 0.19 ^a^ (−24.31) **	7.20 ± 0.47 ^e–i^	8.39 ± 0.50 ^ab^ (14.22) ^ns^	4.88 ± 0.51 ^c–e^	2.17 ± 0.07 ^ghi^ (−55.49) **
Strumicka	3.41 ± 0.30 ^ef^	3.45 ± 0.04 ^f^ (1.42) ^ns^	6.63 ± 0.35 ^g–j^	7.29 ± 0.34 ^a–d^ (8.96) ^ns^	3.86 ± 0.14 ^f–i^	2.06 ± 0.21 ^hi^ (−45.59) **
Tempranas De Machamiel	4.08 ± 0.11 ^c^	4.77 ± 0.04 ^a^ (14.49) **	7.99 ± 0.38 ^c–f^	8.06 ± 0.69 ^a–d^ (0.89) ^ns^	5.83 ± 0.33 ^b^	2.87 ± 0.04 ^bcd^ (−50.79) **
Yavneh	2.85 ± 0.17 ^ghi^	3.07 ± 0.04 ^g^ (7.12) ^ns^	7.20 ± 0.26 ^e–i^	7.65 ± 0.11 ^a–d^ (5.99) ^ns^	3.69 ± 0.33 ^g–j^	2.60 ± 0.17 ^c–f^ (−29.71) *
Zborovicki	2.10 ± 0.06 ^j^	4.17 ± 0.06 ^b^ (49.58) ***	9.71 ± 0.33 ^a^	7.21 ± 0.46 ^a–e^ (−25.71) **	4.99 ± 0.11 ^b–e^	2.63 ± 0.19 ^c–f^ (−47.25) **
Total range	2.04–6.11	2.86–4.77	5.81–9.71	5.78–8.81	2.94–7.84	1.85–3.50
Total mean	3.23	3.77	7.61	7.66	4.62	2.58
CV (%)	27.75	13.59	13.31	9.32	26.02	17.64

Superscript letters in a column indicate significantly different means between faba bean cultivars (*p* < 0.05). Values in parentheses indicate % increase (positive values) or % decrease (negative sign) after dehulling (^ns^ not significant, * *p* < 0.05, ** *p* < 0.01^,^ *** *p* < 0.001).

**Table 3:** Variations of antioxidant activities in whole and dehulled seeds of 22 faba bean cultivars.

Cultivar	DPPH^•^-Scavenging Activity (mg AAE/g)		ABTS^•+^-Scavenging Activity (mg TE/g)		FRAP (mg AAE/g)	
	Whole	Dehulled	Whole	Dehulled	Whole	Dehulled
Abawi# 1	1.08 ± 0.07 ^fgh^	0.99 ± 0.04 ^hi^ (−8.45) ^ns^	6.57 ± 0.29 ^bcd^	6.46 ± 0.40 ^fg^ (−1.66) ^ns^	1.87 ± 0.09 ^d–g^	2.00 ± 0.04 ^d^ (6.80) ^ns^
Aguadulce	1.17 ± 0.07 ^efg^	1.20 ± 0.08 ^fg^ (2.17) ^ns^	6.29 ± 0.20 ^cd^	7.81 ± 0.26 ^cde^ (19.43) **	1.65 ± 0.07 ^f–i^	1.41 ± 0.09 ^f^ (−14.48) *
Algerian	0.85 ± 0.04 ^hi^	0.85 ± 0.04 ^ij^ (0.00) ^ns^	5.87 ± 0.21 ^def^	6.12 ± 0.14 ^gh^ (4.07) ^ns^	1.41 ± 0.07 ^hij^	1.42 ± 0.08 ^f^ (1.08) ^ns^
Alicante	1.32 ± 0.13 ^de^	1.36 ± 0.07 ^de^ (3.46) ^ns^	7.13 ± 0.13 ^ab^	7.62 ± 0.20 ^c-f^ (6.39) *	2.01 ± 0.24 ^de^	1.54 ± 0.07 ^ef^ (−23.35) ^ns^
Ascott	0.54 ± 0.02 ^kl^	1.10 ± 0.02 ^gh^ (50.63) ***	3.07 ± 0.45 ^j^	7.93 ± 0.44 ^cde^ (61.33) **	0.69 ± 0.03 ^k^	1.19 ± 0.02 ^g^ (42.27) ***
Brocal	0.91 ± 0.08 ^hi^	1.05 ± 0.08 ^gh^ (13.71) ^ns^	4.82 ± 0.60 ^gh^	4.57 ± 0.34 ^i^ (−5.19) ^ns^	1.20 ± 0.21 ^j^	1.78 ± 0.22 ^e^ (32.65) ^ns^
Domasna-1	1.26 ± 0.02 ^def^	1.39 ± 0.04 ^de^ (9.65) *	6.02 ± 0.44 ^de^	9.73 ± 0.19 ^ab^ (−26.89) **	1.95 ± 0.08 ^def^	1.62 ± 0.08 ^ef^ (−27.73) **
Domasna-2	1.04 ± 0.09 ^gh^	0.53 ± 0.04 ^k^ (−49.46) **	6.43 ± 0.21 ^bcd^	4.70 ± 0.17 ^i^ (38.14) **	1.59 ± 0.09 ^ghi^	1.15 ± 0.07 ^g^ (−17.08) *
Ethiopia 530	1.07 ± 0.06 ^fgh^	1.49 ± 0.03 ^cd^ (27.87) **	5.13 ± 0.29 ^g^	8.59 ± 0.89 ^cd^ (40.31) **	1.85 ± 0.04 ^d–g^	1.73 ± 0.07 ^e^ (−6.42) ^ns^
Giant Three Seeded	0.78 ± 0.04 ^ij^	1.44 ± 0.15 ^cde^ (46.14) **	6.88 ± 0.23 ^abc^	7.88 ± 0.18 ^cde^ (12.80) **	1.41 ± 0.04 ^hij^	1.56 ± 0.20 ^ef^ (9.48) ^ns^
Large Mazandaran	1.66 ± 0.17 ^b^	1.27 ± 0.03 ^ef^ (−23.52) *	6.38 ± 0.14 ^cd^	8.72 ± 0.46 ^bc^ (26.86) **	2.56 ± 0.16 ^c^	1.48 ± 0.07 ^f^ (−42.25) **
Levens Marschbohne	1.02 ± 0.13 ^gh^	1.57 ± 0.06 ^c^ (34.95) **	3.28 ± 0.36 ^j^	5.94 ± 0.52 ^gh^ (44.77) **	1.39 ± 0.14 ^hij^	1.60 ± 0.05 ^ef^ (12.96) ^ns^
MMR-KJT-2010-K161716	0.69 ± 0.01 ^ijk^	0.87 ± 0.07 ^ij^ (20.05) *	4.28 ± 0.33 ^hi^	6.08 ± 0.75 ^gh^ (29.55) **	1.35 ± 0.02 ^ij^	0.89 ± 0.11 ^h^ (−33.62) **
Muchamiel	1.43 ± 0.17 ^cd^	1.42 ± 0.02 ^cde^ (−1.17) ^ns^	6.97 ± 0.41 ^abc^	6.96 ± 0.25 ^efg^ (−0.20) ^ns^	2.50 ± 0.30 ^c^	2.69 ± 0.08 ^a^ (7.09) ^ns^
NPL-JSW-2003-65	0.62 ± 0.01 ^jk^	0.94 ± 0.01 ^hi^ (33.25) ***	3.71 ± 0.04 ^ij^	8.09 ± 3.04 ^cde^ (54.12)	1.12 ± 0.02 ^j^	1.00 ± 0.12 ^gh^ (−10.42) ^ns^
Pirkkonen	0.35 ± 0.02 ^l^	1.31 ± 0.03 ^ef^ (73.61) ***	7.34 ± 0.13 ^a^	4.55 ± 0.26 ^i^ (−37.94) **	3.62 ± 0.23 ^a^	1.46 ± 0.10 ^f^ (−59.69) ***
Primus	1.53 ± 0.20 ^bc^	2.45 ± 0.10 ^a^ (37.50) **	5.43 ± 0.58 ^efg^	10.34 ± 0.39 ^a^ (47.50) **	2.16 ± 0.27 ^d^	2.49 ± 0.01 ^b^ (13.17) ^ns^
Seville	2.24 ± 0.10 ^a^	2.17 ± 0.20 ^b^ (−3.10) ^ns^	5.41 ± 0.13 ^efg^	7.47 ± 0.05 ^def^ (27.48) ^ns^	3.07 ± 0.13 ^b^	2.29 ± 0.18 ^bc^ (−25.51) **
Strumicka	1.02 ± 0.08 ^gh^	1.09 ± 0.09 ^gh^ (7.05) ^ns^	5.25 ± 0.45 ^fg^	6.61 ± 0.26 ^fg^ (20.58) *	1.21 ± 0.13 ^j^	1.19 ± 0.06 ^g^ (−2.02) ^ns^
Tempranas De Machamiel	2.09 ± 0.13 ^a^	2.13 ± 0.03 ^b^ (1.76) ^ns^	6.37 ± 0.22 ^cd^	7.46 ± 0.78 ^def^ (14.63) ^ns^	3.25 ± 0.11 ^b^	2.30 ± 0.02 ^bc^ (−29.03) **
Yavneh	1.00 ± 0.10 ^gh^	0.72 ± 0.04 ^j^ (−28.15) *	6.49 ± 0.10 ^bcd^	5.30 ± 0.39 ^hi^ (−18.41) *	1.69 ± 0.14 ^e–h^	1.56 ± 0.07 ^ef^ (−7.65) ^ns^
Zborovicki	0.79 ± 0.04 ^ij^	2.07 ± 0.08 ^b^ (61.97) ***	3.10 ± 0.28 ^j^	8.59 ± 0.36 ^cd^ (63.93) ***	1.13 ± 0.10 ^j^	2.19 ± 0.09 ^cd^ (48.26) ***
Total range	0.35–2.24	0.53–2.45	3.07–7.34	4.55–10.34	0.69–3.62	0.89–2.69
Total mean	1.11	1.34	5.56	7.16	1.85	1.66
CV	40.94	36.38	23.50	21.81	39.72	28.48

Superscripts in a column show significantly different means (*p* < 0.05). Values in parentheses indicate a % increase (positive values) or a % decrease (negative sign) after dehulling (^ns^ not significant, * *p* < 0.05, ** *p* < 0.01, *** *p* < 0.001).

**Table 4:** Factor loading values and contributions of variables in the first four principal components.

Parameter	PC1		PC2		PC3		PC4	
	FL	%	FL	%	FL	%	FL	%
Crude fiber content	0.28	1.49	0.89	19.57	0.19	1.26	−0.01	0.02
Dietary fiber content	0.32	1.94	0.86	18.46	0.14	0.69	−0.05	0.25
Crude protein	0.06	0.07	−0.70	12.14	−0.31	3.46	0.43	15.44
Crude fat	−0.38	2.80	−0.58	8.48	−0.04	0.06	−0.47	18.75
Palmitic acid	0.88	15.09	−0.29	2.09	0.11	0.39	−0.18	2.85
Stearic acid	0.33	2.09	0.28	1.94	−0.01	0.01	0.67	38.11
Oleic acid	−0.88	15.29	0.29	2.04	0.19	1.24	−0.02	0.04
Linoleic acid	0.80	12.41	−0.29	2.11	−0.26	2.45	0.05	0.25
Linolenic acid	0.68	9.16	−0.19	0.92	0.06	0.11	−0.43	15.92
TSFA	0.94	17.26	−0.19	0.87	0.10	0.32	0.04	0.12
TUFA	−0.94	17.26	0.19	0.87	−0.10	0.32	−0.04	0.12
Total phenolic content	−0.19	0.73	−0.47	5.43	0.77	20.90	−0.03	0.07
Total tannin content	0.44	3.77	0.64	10.03	0.46	7.55	−0.08	0.55
Total saponin content	−0.09	0.16	0.04	0.04	0.41	5.97	0.16	2.16
DPPH^•^-scavenging activity	−0.13	0.35	−0.34	2.91	0.74	19.38	0.20	3.35
ABTS^•+^-scavenging activity	−0.03	0.02	−0.70	12.05	0.46	7.34	0.15	1.89
Ferric-reducing antioxidant power	0.08	0.13	0.04	0.05	0.90	28.55	−0.04	0.11
Eigenvalue	5.11		4.02		2.85		1.18	
Variability (%)	30.08		23.66		16.76		6.97	
Cumulative variance (%)	30.08		53.73		70.49		77.46	

FL: Factor loading; PC: Principal component; TSFA: Total saturated fatty acid; TUFA: Total unsaturated fatty acid.

## Data Availability

All the data related to this study are incorporated in the manuscript and Supplementary Materials. Further inquiries can be directed to the corresponding author.

## Supplementary Materials

The following supporting information can be downloaded at: https://www.mdpi.com/article/10.3390/foods12224063/s1, Figure S1: Variations of metabolite contents, fatty acids, and antioxidant activities between dehulled and whole seeds in the whole population of faba bean cultivars. Different letters on boxplots indicate significantly different means (*p* < 0.05). ABTS: ABTS^•+^-scavenging activity; CFC: Crude fiber content; DFC: Dietary fiber content; DPPH: DPPH^•^-scavenging activity; FRAP: Ferric-reducing antioxidant power; TPC: Total phenolic content; TSC: Total saponin content; TSFA: Total saturated fatty acid; TTC: Total tannin content; TUFA: Total unsaturated fatty acid; Table S1: Total fiber, total protein, and total fat contents (% dry weight basis) and effect of dehulling in 22 faba bean cultivars.
