# Supplementary material for: Nutrient Levels, Bioactive Metabolite Contents, and Antioxidant Capacities of Faba Beans as Affected by Dehulling
Source: Foods. 2023 Nov 8;12(22):4063. doi: 10.3390/foods12224063 (PMC10670910; doi:10.3390/foods12224063)
Supplement: Supplementary file 1 [file foods-12-04063-s001.zip › foods-2648527-supplementary.pdf]

Supplementary material

Nutrient Levels, Bioactive Metabolite Contents, and Antioxidant Capacities of Faba Beans as Affected by Dehulling

Yu-Mi Choi[1], Hyemyeong Yoon[1], Myoung-Jae Shin[1], Sukyeung Lee[2], Jungyoon Yi[1], Young-ah Jeon[1], Xiaohan Wang[1], Kebede Taye Desta[1,*]

[1] National Agrobiodiversity Center, National Institute of Agricultural Sciences, Rural Development Administration, Jeonju 54874, Republic of Korea
[2] International Technology Cooperation Center, Technology Cooperation Bureau, Rural Development Administration, Jeonju 54875, Republic of Korea
* Correspondence: kebedetdesta@korea.kr

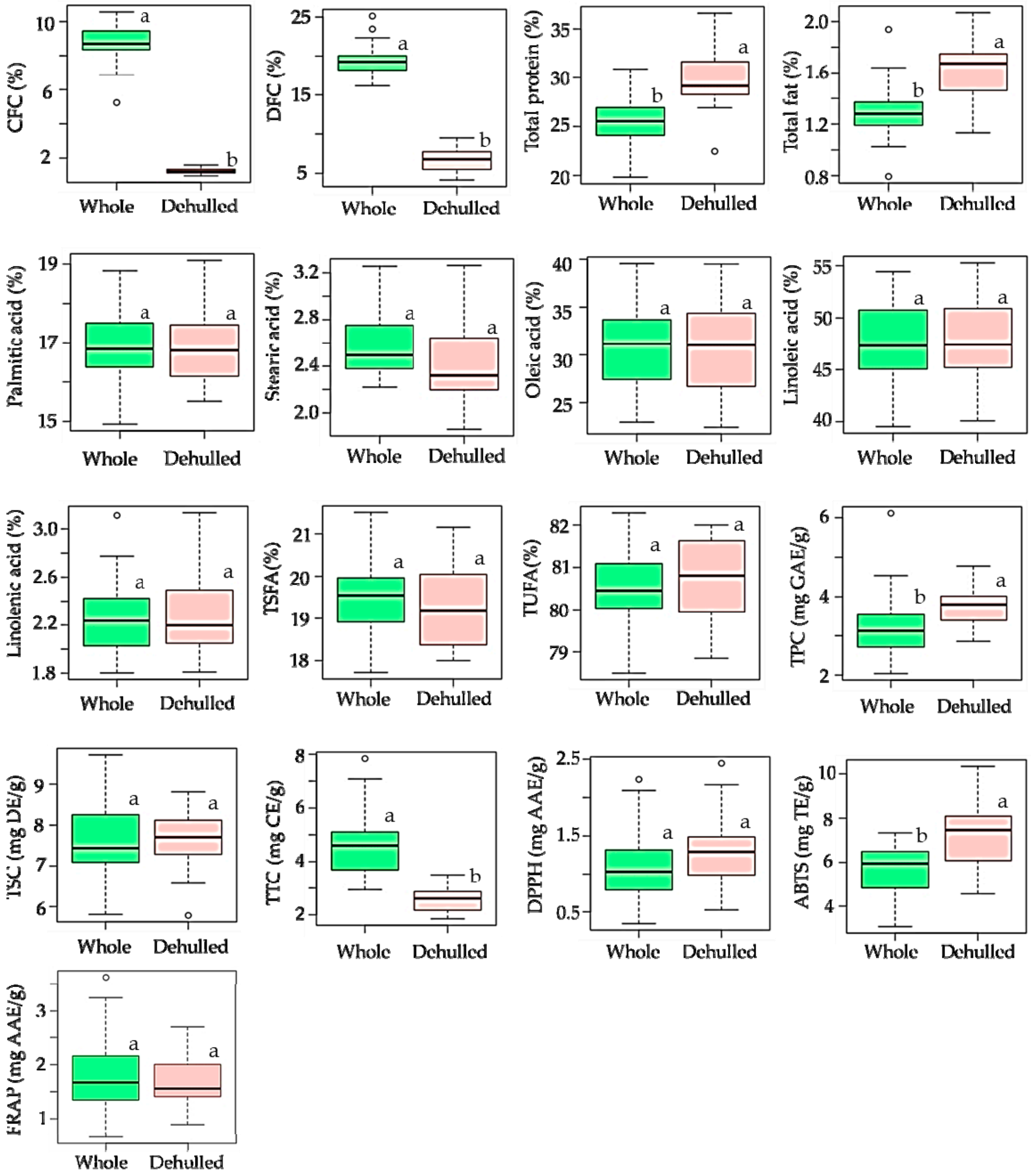

Figure S1. Variations of metabolite contents, fatty acids and antioxidant activities between dehulled and whole seeds in the whole population of faba bean cultivars. Different letters on boxplots indicate significantly different means ($p < 0.05$). ABTS: $ABTS^{\bullet+}$ scavenging activity; CFC: Crude fiber content; DFC: Dietary fiber content; DPPH: $DPPH^{\bullet}$ scavenging activity; FRAP: Ferric reducing antioxidant power; TPC: Total phenolic content; TSC: Total saponin content; TSFA: Total saturated fatty acid; TTC: Total tannin content; TUFA: Total unsaturated fatty acid.

Table S1. Total fiber, total protein, total fat, double bond index and fatty acid ratio, and effect of dehulling on each in 22 faba bean cultivars.

Cultivar	Source	Crude fiber (%)		Dietary fiber (%)		Total protein (%)		Total fat (%)		DBI		ΣTUFA/ΣTSFA	
		W	D	W	D	W	D	W	D	W	D	W	D
Abawi# 1	Peru	9.52^{bc}	1.188 ^{fg}	21.760 ^{bcd}	7.393 ^{bcde}	24.177 ^{i}	27.977 ^{j}	1.283 ^{efg}	1.624 ^{bcd}	134.68	137.15	4.29	4.36
Aguadulce	Spain	6.88^{h}	1.128 ^{gh}	19.346 ^{defghi}	7.245 ^{bcde}	28.618 ^{b}	33.523 ^{c}	1.334 ^{def}	1.676 ^{bcd}	135.17	135.63	3.77	3.83
Algerian	Iran	9.84^{b}	1.410 ^{bc}	23.455 ^{ab}	9.504 ^{a}	28.147 ^{c}	34.196 ^{b}	1.443 ^{cd}	1.670 ^{bcd}	132.46	132.48	4.15	4.22
Alicante	Israel	8.35^{e}	1.093 ^{h}	17.428 ^{ghij}	4.335 ^{hi}	28.631 ^{b}	32.549 ^{d}	1.221 ^{efg}	1.690 ^{bcd}	130.35	131.87	4.01	4.06
Ascott	France	7.61^{fg}	1.460 ^{b}	18.199 ^{fghij}	7.967 ^{bc}	26.475 ^{e}	30.733 ^{f}	1.277 ^{efg}	1.584 ^{cde}	131.01	130.92	4.53	4.44
Brocal	Spain	9.85^{b}	1.318 ^{de}	22.335 ^{bc}	7.998 ^{bc}	24.056 ^{i}	26.912 ^{l}	1.348 ^{de}	1.416 ^{fg}	129.11	128.32	3.84	4.30
Domasna-1	Macedonia	8.39 ^{e}	1.192 ^{fg}	20.031 ^{cdef}	6.635 ^{def}	26.941 ^{d}	31.551 ^{e}	1.252 ^{efg}	1.409 ^{fg}	130.08	130.30	4.07	4.48
Domasna-2	Macedonia	8.71 ^{de}	1.319 ^{de}	19.955 ^{cdefg}	7.755 ^{bcd}	26.116 ^{f}	31.002 ^{f}	1.031 ^{h}	1.461 ^{efg}	133.22	136.17	4.08	4.10
Ethiopia 530	Israel	9.53 ^{bc}	1.055 ^{hi}	19.554 ^{defg}	7.014 ^{cde}	24.559 ^{h}	28.975 ^{hi}	1.640 ^{b}	1.764 ^{b}	131.90	132.30	4.44	4.56
Giant Three Seeded	Japan	8.74 ^{de}	1.174 ^{fg}	20.012 ^{cdef}	4.142 ^{i}	24.845^{gh}	28.814 ^{i}	1.938 ^{a}	1.693 ^{bcd}	127.09	131.22	4.61	4.48
Large Mazandaran	Iran	9.16 ^{cd}	1.052 ^{hi}	19.461 ^{defgh}	5.301 ^{ghi}	23.424 ^{j}	27.527 ^{k}	1.190 ^{fg}	1.954 ^{a}	136.26	136.69	3.70	3.73
Levens Marschbohne	Germany	7.82 ^{f}	1.393 ^{bcd}	16.947 ^{hij}	6.794 ^{cde}	25.002 ^{g}	28.923 ^{hi}	1.139 ^{gh}	1.375 ^{g}	139.96	140.38	4.18	4.04
MMR-KJT-2010-K161716	Myanmar	5.24 ^{i}	1.592 ^{a}	16.845 ^{ij}	4.844 ^{hi}	26.404 ^{ef}	29.938 ^{g}	1.332 ^{def}	1.748 ^{bc}	132.87	134.28	4.03	4.03
Muchamiel	Spain	10.56 ^{a}	1.348 ^{cd}	25.117 ^{a}	8.449 ^{ab}	26.072 ^{f}	31.654 ^{e}	1.527 ^{bc}	1.667 ^{bcd}	136.35	137.84	3.89	3.93
NPL-JSW-2003-65	Nepal	8.56 ^{e}	1.555 ^{a}	19.180 ^{efghi}	7.785 ^{bcd}	28.585 ^{b}	33.474 ^{c}	1.025 ^{h}	1.977 ^{a}	136.61	135.48	3.65	3.95
Pirkkonen	Sweden	8.68 ^{de}	0.958 ^{j}	18.570 ^{efghij}	4.932 ^{hi}	24.959 ^{g}	29.176 ^{h}	1.371 ^{de}	1.414 ^{fg}	131.46	131.68	4.12	4.46
Primus	Hungary	8.68 ^{de}	1.351 ^{cd}	16.165 ^{j}	7.260 ^{bcde}	30.904 ^{a}	36.611 ^{a}	0.794 ^{i}	1.132 ^{h}	132.25	131.90	4.11	3.90
Seville	UK	8.67 ^{de}	1.249 ^{ef}	18.328 ^{efghij}	6.774 ^{cde}	24.093 ^{i}	27.477 ^{k}	1.224 ^{efg}	2.068 ^{a}	125.24	125.81	4.34	4.44
Strumicka	Macedonia	9.45 ^{bc}	1.126 ^{gh}	20.854 ^{cde}	6.741 ^{cde}	23.446 ^{j}	28.300 ^{j}	1.328 ^{def}	1.999 ^{a}	131.28	131.61	4.20	4.36
Tempranas De Machamiel	Spain	9.23 ^{cd}	1.205 ^{fg}	18.865 ^{efghi}	6.706 ^{cde}	19.829 ^{k}	22.467 ^{m}	1.588 ^{b}	1.701 ^{bcd}	129.07	128.94	4.13	4.20
Yavneh	Israel	7.10 ^{gh}	0.996 ^{ij}	17.773 ^{fghij}	6.367 ^{efg}	26.405 ^{ef}	29.115 ^{hi}	1.030 ^{h}	1.544 ^{def}	135.71	136.25	4.11	3.99

Zborovicki	Czech Republic	9.25 [cd]	1.252 [ef]	18.480 [efghij]	5.492 [fgh]	23.836 [i]	28.264 [j]	1.220 [efg]	1.690 [bcd]	127.79	128.96	4.64	4.47
Range		5.24-10.56	0.96-1.59	16.17-25.12	4.14-9.50	19.83-30.90	22.47-36.61	0.79-1.94	1.13-2.07	125.24-139.96	125.81-140.38	3.65-4.64	3.73-4.56
Total mean		8.63	1.25	19.48	6.70	25.71	29.96	1.30	1.65	132.27	133.01	4.13	4.20
CV (%)		13.25	13.53	10.96	20.11	0.09	9.88	18.19	13.52	2.63	2.64	6.41	5.83

Different superscript letter in a column indicate significantly different means ($p < 0.05$).
CV: Coefficient of variation, D: Dehulled, DBI: Double bond index, TSFA: Total saturated fatty acid, TUFA: total unsaturated fatty acid, UK: United Kingdom, W: Whole.
